# 

**Published:** 1916-05

**Authors:** 


### Contents

Event and Comment .............................. 201
Conductive Anesthesia .......................... 204
New Method of Constructing Full Dentures......... 224
What Should Be the Fee for a Denture?............ 227
Indents ........................................ 233
Announcements .................................. 246
Obituary ....................................... 247
Bibliography ................................... 249

